# Development and Experimental Evaluation of a 3D Vision System for Grinding Robot

**DOI:** 10.3390/s18093078

**Published:** 2018-09-13

**Authors:** Shipu Diao, Xindu Chen, Jinhong Luo

**Affiliations:** 1 School of Electromechanical Engineering, Guangdong University of Technology, Guangzhou 510006, China; sipoudiu@gmail.com; 2 Guangdong Provincial Key Laboratory of Computer Integrated Manufacturing System, Guangzhou 510006, China; cimsrobot@gmail.com

**Keywords:** grinding robot, 3D vision system, machining target, point cloud

## Abstract

If the grinding robot can automatically position and measure the machining target on the workpiece, it will significantly improve its machining efficiency and intelligence level. However, unfortunately, the current grinding robot cannot do this because of economic and precision reasons. This paper proposes a 3D vision system mounted on the robot’s fourth joint, which is used to detect the machining target of the grinding robot. Also, the hardware architecture and data processing method of the 3D vision system is described in detail. In the data processing process, we first use the voxel grid filter to preprocess the point cloud and obtain the feature descriptor. Then use fast library for approximate nearest neighbors (FLANN) to search out the difference point cloud from the precisely registered point cloud pair and use the point cloud segmentation method proposed in this paper to extract machining path points. Finally, the detection error compensation model is used to accurately calibrate the 3D vision system to transform the machining information into the grinding robot base frame. Experimental results show that the absolute average error of repeated measurements at different locations is 0.154 mm, and the absolute measurement error of the vision system caused by compound error is usually less than 0.25 mm. The proposed 3D vision system could easily integrate into an intelligent grinding system and may be suitable for industrial sites.

## 1. Introduction

Surface grinding is usually the last step in the manufacturing process of workpieces with high surface quality requirements. Due to the manufacturing costs before the grinding process, any unqualified workpiece after grinding means increasing economic losses. Although it tends to increase the level of automation, the grinding of surfaces with complex geometries is mainly done manually [1,2]. Industrial robots make it ideal for grinding the workpiece’s surface [3,4,5,6,7]. However, current grinding robots are usually only able to machine the fixed-position target by manually teaching machining instructions, which are not available for changes in the position of the machining target on the workpiece. Therefore, it is necessary to study the robot vision system for automatic positioning and measurement of the machining target. Which not only can solve the processing problem when the machining target position changes, but also can improve the machining efficiency and intelligent level of the grinding system.

To improve the ability of robots to perform tasks, in recent years, scientists around the world have added different vision systems to their robots. In general, the function of the robot’s vision system is mainly focused on the pose measurement of the workpiece and the guidance of the robot movement. On the one hand, when the robot vision system is used for three-dimensional pose measurement of workpieces, Lindner et al. [8] presented a novel robot vision system, which uses dynamic laser triangulation, to determine three-dimensional (3D) coordinates of an observed object. According to industrial assembly line sorting activities, the sorting system of an industrial robot was designed by Qin et al. [9] based on vision detection. The workpiece recognition method is used to identify the workpiece and calculate the centre coordinates of the workpiece which are unified to the robot frame and communicate with the robot. The robot can accurately grasp and sort the workpiece. Zhang et al. [10] proposed an approach of the object-oriented vision-guided robot that video segmentation, tracking and recognition are used to guide the robot to reduce the complexity of 3D object detection, recognition and pose estimation. Montironi et al. [11] presented an adaptive strategy for automatic camera placement in a 3-dimensional space during a robotised vision-based quality control. On the other hand, when the robot vision system is used to guide the robot movement, Šuligoj et al. [12] proposed a method of frame relative displacement and described a working multiagent robot application that can be used for tracking, tooling or handling operations with the use of stereo vision in an unstructured laboratory environment. One robot arm carries a stereo vision camera system, and the other robot has a marker that is used for navigation between the robot and the object of interest. Although this method achieves its purpose, the overall hardware cost is still relatively high. Therefore, some researchers have proposed a lower cost solution. To improve the operational efficiency of robot-based shoe manufacturing, a method of shoe-groove tracking based on the industrial robot was presented by Wu et al. [13]. The presented approach mainly consists of two steps: reconstruction of three-dimensional point cloud and feature curve extraction. Also, some researchers have researched obtaining navigation data based on point cloud analysis. Gonçalves et al. [14] presented the algorithms and software tools developed for US-based orthopaedic surgery navigation. For navigation, the system, during surgery, acquires a 3D US bone surface from a sequence of US images. This bone surface will then be registered to the pre-operative bone model, for precise knowledge of the bone position and orientation. This registration is performed in two steps: global registration and locally register.This method based on point cloud analysis to obtain navigation data is feasible. Different from the above use of the vision system for the robot to solve a specific problem, Pérez et al. [15] gave a comparative review of different machine vision techniques for robot guidance is presented. Moreover, the work analyses the accuracy, range and weight of the sensors, safety, processing time and environmental influences. These scientists have been successfully added different vision systems to the robot to solve many problems. This provides a reference for the robot to choose an appropriate vision system solution. Through the above works of literature, we can find that adding a vision system to the robot can solve some specific problems, but adding a suitable vision system to the grinding robot is still a challenge because it not only needs to solve the problem of 3D measurement but also solve the problem of guiding the robot.

However, some scholars have studied the visual system of grinding robots. The purpose of their research is usually to improve the processing efficiency of grinding robots. Their research contents are as follows: Dieste et al. [16] proposed a novel grinding system based on robotics and artificial vision. The application of this novel system has allowed reducing the failed parts due to finishing process down to zero percent from 28% of rejected parts with a manual grinding process. The reduction in process time consuming, and amount of scrapped parts has reduced the energy consumption up to 30% in the finishing process, and 20% in the whole manufacturing process for an injection moulded aluminium part for the automotive industry with high production volumes. In the study of Hosseininia et al. [17], Machine vision technology and image processing techniques are employed, implemented and integrated with the current robotic system to detect the pose (position and orientation) of biscuits, hence guiding the robot arm to perform its grinding operation at the correct and most efficient pose. Princely et al. [18] proposed a Vision-Guided Robotic System (VGRS) methodology for the deburring of workpieces to eliminate the weakness of “Teach” or “Offline” programming methodologies. In this proposed system the shape of the two-dimensional workpiece is acquired for each workpiece, and the robot-language program is generated automatically from the workpiece shape data and finishing condition data. Ji et al. [19] described an autonomous robot developed to implement wall grinding work. It is designed to implement wall grinding work which belongs to the fundamental wall finishing works and is harmful to workers due to the accompanied dust. The proposed robot system consists of a structured light-based 3D scanner, and a robot arm (UR5, Universal Robots, Odense, Denmark) fixed on a mobile platform. The 3D scanner is adapted for recognising the defects on the wall surface and measuring its roughness. These scholars have been added visual systems to their robots, which significantly improve the level of intelligence of robots. However, the research on installing 3D scanners on the middle joint of a robot is rare because the difficulty of such solution is needed to overcome the problem of how to obtain machining information through point cloud analysis and how to accurately calibrate the robot 3D vision system.

The objective of this article is to develop and evaluate a 3D vision system for grinding robots, and its functions will mainly include the following two points: (1) High-precision positioning and measurement of the machining target; (2) Transformation of the machining information described by the 3D scanner frame into the robot base frame. Therefore, the grinding robot 3D vision system needs not only to be able to detect the machining target with high accuracy to obtain machining information but also to be able to transform machining information into the robot base frame with high accuracy. Besides, the 3D scanner is mounted on the robot’s fourth joint, so the data processing of the 3D vision system proposed in this paper includes point cloud acquisition and preprocessing, point cloud registration, machining information extraction, and coordinate transformation of machining information.

The main contribution of this paper is the following:

- We proposed a grinding robot 3D vision system to detect the machining target automatically.
- To improve the speed and accuracy of registration, we use the voxel grid filter and fast point feature histogram (FPFH) to preprocess the sample point cloud and the test point cloud. To improve the registration accuracy of the point cloud pair, we use the combination of the sampling consistent initial registration algorithm (SAC-IA) and iterative closest point algorithm (ICP).
- We use FLANN to search out the difference point cloud from the precisely registered point cloud pair and use the point cloud segmentation method proposed in this paper to extract machining path points from the difference point cloud. The key point of the point cloud segmentation is as follows: (1) Calculate the minimum bounding box. (2) Rasterize the bounding box and find the centroid of the point cloud in each three-dimensional grid. (3) Use neighbour search to search for the nearest point to the centroid from the test point cloud.
- We construct a detection error compensation model for the vision system and use a multi-point constraint method and a high-precision laser tracker to accurately calculate the error compensation amount and the transformation matrix of the scanner frame to the robot base frame.

The rest of this paper is organised as follows. Section 2 describes the hardware architecture of the grinding robot 3D vision system. Section 3 introduces the mathematical basis of the grinding robot 3D vision system, including the algorithms used in the data processing of the 3D vision systems and the calibration methods for the grinding robot 3D vision system. Section 4 introduces the data processing of the grinding robot 3D vision system, including point cloud acquisition and preprocessing, point cloud registration, and machining information extraction and frame transformation. Section 5 is the experimental results and the discussion of the effects of related factors on the detection accuracy of the vision system. Section 6 presents the conclusion and suggestions for future work.

## 2. The Hardware Architecture of the Grinding Robot 3D Vision System

**Figure 1:**
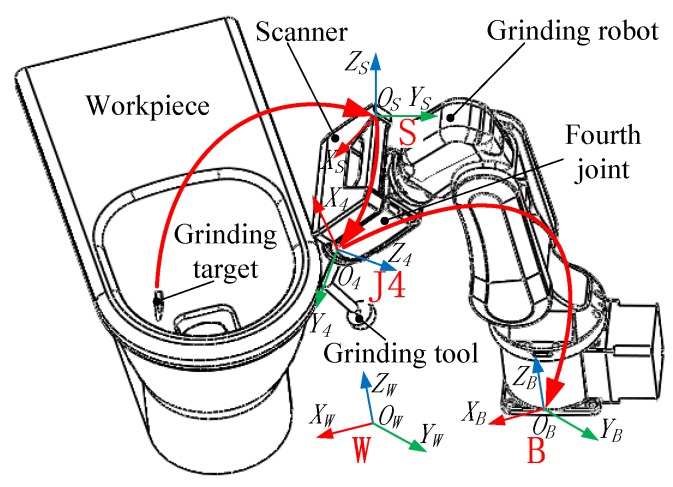
Schematic diagram of the grinding robot 3D vision system.

Figure 1 shows the hardware architecture of the grinding robot 3D vision system proposed in this paper, including the grinding robot and the 3D scanner fixed on the grinding robot’s fourth joint. The frames involved include the world frame (W), the robot base frame (B), the robot’s fourth joint frame (J4), and the scanner frame (S). Where B and W are usually coincident.

Suppose the coordinate value of a machining path point on the machining target in the scanner frame *S* is XS, and the coordinate value in the robot base frame *B* is XB, then

(1) XB=TSJ4J4BTSX

Here TJ4B is the transformation matrix between the robot’s fourth joint frame and the robot base frame, and TSJ4 is the transformation matrix between the scanner frame and the robot’s fourth joint frame.

From the above analysis, it can be known that the data flow direction of the machining information of the 3D vision system in different frames is: S→J4→B. Therefore when we use the 3D vision system to detect the machining target, the first task is to extract the machining path points from the machining target, and the second task is to transform the machining information from the scanner frame to the robot base frame.

## 3. The Mathematical Basis for the Grinding Robot 3D Vision System

Section 2 introduces the hardware architecture of the grinding robot 3D vision system. In this section, the mathematical basis of the 3D vision system will be introduced, including downsample point cloud using VoxelGrid filter, calculate FPFH, coarse registration of point cloud pair based on SAC-IA, accurate registration of point cloud pair based on ICP algorithm, using FLANN to search for difference point cloud, extract machining path points from difference point cloud, and accurate calibration of the grinding robot 3D vision system based on error compensation model.

### 3.1. Downsample Point Cloud Using Voxel Grid Filter

To improve the registration speed of the test point cloud and the sample point cloud, these point clouds need to be downsampled. In this paper, we use a voxel grid filter [20,21] to downsample the point cloud. When the point cloud is sampled by the voxel grid filter, a plurality of three-dimensional voxel grids are created by inputting point cloud data, and then all the points in the voxel are approximated by centroid points of all the points in each voxel. In the process of creating a three-dimensional voxel grid, the number of voxels m is inversely proportional to the setting value of the voxel volume v. When calculating the centroid ($X_{ct}$, $Y_{ct}$, $Z_{ct}$) of each three-dimensional voxel, and we use the following equation:

(2) $$\left\{\begin{array}{c} X_{ct}=\sum_{i=1}^{g}x_{i}/g\\ Y_{ct}=\sum_{i=1}^{g}y_{i}/g\\ Z_{ct}=\sum_{i=1}^{g}z_{i}/g \end{array}\right.$$

Here, *g* is the number of points in the current voxel, and ($x_{i}$, $y_{i}$, $z_{i}$) (i∈[1,g]) is a three-dimensional coordinate value of a point within the current voxel. Using Equation (2) to process all three-dimensional voxels we can complete the downsampling of the point cloud.

### 3.2. Calculate FPFH

Registration of point cloud pair relies on feature descriptor. Before registering the point cloud, we need to calculate the point feature descriptor of the point cloud. This paper uses the FPFH [22,23,24] descriptor. The procedure of calculating FPFH can be described as follows:

Step 1. Calculate the simple point feature histograms (SPFH) of the current query point $P_{q}$. In this step, we need to calculate a tuple between the current query point $P_{q}$ and the neighbourhood $P_{k}$. Assuming that the normals corresponding to the current query point $P_{q}$ and the neighbourhood $P_{k}$ are $n_{q}$ and $n_{k}$, respectively, the uvw frame can be obtained.

(3) $$\begin{array}{c} u=n_{q}\\ v=(P_{k}-P_{q})\times u\\ w=u\times v \end{array}$$

Then the deviation between normal $n_{q}$ and $n_{k}$ can be represented by a set of angles, i.e.,

(4) α=v·nkϕ=u·(Pk−Pq)(Pk−Pq)ddθ=arctan(w·nk,u·nk)

Here, *d* is the Euclidean distance between two points $P_{q}$ and $P_{k}$, that is, $d={∥P_{k}-P_{q}∥}_{2}$.

Step 2. Calculate FPFH of $P_{q}$. In this step, we need to re-determine the k-neighbourhood of each point and use the neighbouring SPFH values to calculate the FPFH of $P_{q}$, i.e.,

(5) $$\mathrm{FPFH}(P_{q})=\mathrm{SPFH}(P_{q})+\frac{1}{k}\sum_{i=1}^{k}\frac{1}{w_{k}}\cdot \mathrm{SPFH}(P_{k})$$

Here, the weight $w_{k}$ represents the distance between the query point $P_{q}$ and its neighbouring point $P_{k}$ in the given metric space.

### 3.3. Coarse Registration of the Point Cloud Pair Based on SAC-IA

To accurately register the point cloud pair, it is necessary to first coarsely register point cloud pair. In this paper, SAC-IA [24] is used to finish the coarse registration. Performing a coarse registration requires the following steps.

Step 1. Select *s* sample points from point cloud *P* while making sure that their pairwise distances are greater than a user-defined minimum distance $d_{min}$.

Step 2. For each of the sample points, find a list of points in *Q* whose histograms are similar to the sample points’ histogram. From these, select one randomly which will be considered that sample points’ correspondence.

Step 3. Compute the rigid transformation defined by the sample points and their correspondences and compute an error metric for the point cloud that computes the quality of the transformation. The error metric here is determined using a Huber penalty measure $L_{h}$:

(6) $$L_{h}\left(e_{i}\right)=\left\{\begin{array}{c} 0.5e_{i}^{2}\\ 0.5t_{e}\left(2∥e_{i}∥-t_{e}\right) \end{array}\right.$$

In Equation (6), $e_{i}$ is used to describe the difference between the observed value and the predicted value, and $t_{e}$ is the hyperparameter used to control the error.

These three steps are repeated, and the transformation that yielded the best error metric is stored and used to align the partial views roughly. Finally, non-linear local optimisation is applied using a Levenberg-Marquardt algorithm [25].

### 3.4. Accurate Registration of Point Cloud Pair Based on ICP

To accurately register the point cloud pair, this paper uses ICP [26] to estimate the transformation matrix accurately. The ICP algorithm can be stated: 1. The point set *P* with $N_{p}$ points from the data shape and the model shape *X* is given. 2. The iteration is initialized by setting $P_{0}=P$, ${\vec{q}}_{0}={\left[1,0,0,0,0,0,0\right]}^{t}$ and k=0. The registration vectors are defined relative to the initial data set $P_{0}$ so that the final registration represents the complete transformation. Steps 1, 2, 3, and 4 are applied until convergence within a tolerance τ.

Step 1. Compute the closest points: $Y_{k}=C\left(P_{k},X\right)$.

Step 2. Compute the registration: $\left({\vec{q}}_{k},d_{k}\right)=Q\left(P_{0},Y_{k}\right)$.

Step 3. Apply the registration: $P_{k+1}={\vec{q}}_{k}\left(P_{0}\right)$.

Step 4. Terminate the iteration when the change in mean-square error falls below a preset threshold τ>0 specifying the desired precision of the registration: $d_{k}-d_{k+1}<\tau$.

### 3.5. Search for Difference Point Cloud Using FLANN

To search out the difference point cloud from the precisely registered point cloud pair, FLANN [27] is used in this paper. FLANN package is a ready library to work with, and the fast library for approximate nearest neighbours package is proposed by Muja and David based on existing research results [28,29,30,31,32], which includes hierarchical k-means trees and multiple randomised kd-trees. Compared to linear search, FLANN can accelerate the matching of high-dimensional vectors by up to several orders of magnitude.

By considering the algorithm itself as a parameter of a generic nearest neighbour search routine, the problem is reduced to determining the parameters that give the best solution. This is an optimisation problem in the parameter space. We can choose the best nearest neighbour algorithm and the optimum parameters in a two-step approach:

Step 1. Use $\left\{1,4,8,16,32\right\}$ as the number of random kd-trees, $\left\{16,32,64,128,256\right\}$ as the branching factor for the k-means tree and $\left\{1,5,10,15\right\}$ as the number of k-means iterations.

Step 2. Use the Nelder-Mead downhill simplex method to further locally explore the parameter space and fine-tune the best parameters obtained in Step 1.

### 3.6. Extract Machining Path Points From Difference Point Cloud

In this paper, machining path points are extracted by segmenting the difference point cloud. The procedure of extracting machining path points from the difference point cloud can be described as follows:

Step 1. Calculate the minimum bounding box of the current difference point cloud [33,34].

Step 2. Convert the minimum bounding box obtained in Step 1 to the Corp-Box, divide the difference point cloud along the longest side direction into multiple parts, and then use the Crop-Box filter [35] to divide the difference point cloud.

Step 3. Store the point cloud of each small box as a vector type of the point cloud, and then calculate the centroid of each point cloud.

Step 4. Use nearest neighbour search to find the closest point to the centroid on the test point cloud in Step 3 as the machining path point.

### 3.7. Accurate Calibration of the Grinding Robot 3D Vision System Based on Error Compensation Model

To accurately transform the machining information described by the scanner frame into the robot base frame description, this paper constructs an error compensation model to accurately calibrate the grinding robot 3D vision system and obtain the compensated frame transformation matrix. In the calibration process, we first use the three-point method for coarse calibration and then use the error compensation model for accurate calibration. This calibration method is similar to [36], but the difference is hardware architecture, use occasion, coarse calibration method, and error compensation model.

We need the robot’s kinematics model when calibrating the vision system. Robot calibration needs to ensure enough parameters to describe all possible movements. According to the improved DH parameter method, the transformation relationship between adjacent link frames can be represented by four motion errors, namely the link length $a_{i-1}$, the link angle $\alpha_{i-1}$, the link offset $d_{i}$, and the joint angle $\theta_{i}$. However, the disadvantage of the DH parameter method is that when the two adjacent joints are nominally parallel, a slight change in the attitude of the joint axis will cause a massive change in the DH parameter. Therefore, this paper uses the MDH parameter method to construct the kinematics model of the robot. This method is based on the DH motion model to overcome the defects of the DH parameter method and introduce the additional rotation item $\beta_{i}$. The second and third joints of the industrial robot used in this paper are parallel. Therefore, the MDH method is used to model the adjacent joints. That is, the rotation parameter $\Delta \beta_{2}$ around the Y-axis is introduced at the third joint. For other joints, use the modified DH method for modelling.

The coarse calibration process can be described as follows:

Step 1. Keep an appropriate orientation robot, place the target sphere at three different positions *o*, *a*, *b* in the measurement space, and obtain the spherical point cloud corresponding to the sphere in the scanner frame, and read the coordinate values $o_{B}$, $a_{B}$, $b_{B}$ of the corresponding position in the robot base frame from the laser tracker. Then use the random sample consensus (RANSAC) [37] to segment the spherical point cloud to get the coordinates of the centre of the sphere $o_{S}$, $a_{S}$, $b_{S}$.

Step 2. Use $o_{S}$, $a_{S}$, $b_{S}$ to create the workpiece frame $P_{S}$ relative to the scanner frame, and use $o_{B}$, $a_{B}$, $b_{B}$ to establish the workpiece frame $P_{B}$ relative to the robot base frame.

Step 3. Calculate the coordinate transformation matrix between the robot’s fourth joint frame and the robot base frame T4B:

(7) T4B=T211BT32T43T

Then, calculate the coordinate transformation matrix of the scanner frame to the robot’s fourth joint frame TS4:

(8) TinitialS4=T−14BPBPS−1

Equation (8) is the result of the coarse calibration.

The accurate calibration process can be described as follows:

Step 1. Construct a detection error model for the grinding robot 3D vision system. When the high-order terms are ignored the actual position $X_{i}^{R}$ of the sphere centre in the robot’s base frame is:

(9) XBiR=(Ti4B+d4BTi)(Ts4+dS4T)XSi≈(Ti4BTS4+Ti4BdS4T+d4BTiTS4)XSi=XBi+Ti4BdS4TXSi+d4BTiTS4XSi

In Equation (9), $d_{S}^{4}T$ represents the error of the transformation matrix of the scanner frame to the robot’s fourth joint frame, and $d_{4}^{B}T$ represents the error of the transformation matrix of the robot’s fourth joint frame to the robot base frame.

The second term of Equation (9) can be transformed into:

(10) Ti4BdS4TXSi=Ti4B1000(TS4XSi)z−(TS4XSi)y010−(TS4XSi)z0(TS4XSi)x001(TS4XSi)y−(TS4XSi)x0dx1dy1dz1δx1δy1δz1

Assuming that the second 3×6 matrix in Equation (10) is $P_{i}$ and the third 6×1 matrix is $Er_{1}$, then Equation (10) can be transformed into:

(11) Ti4BdS4TXSi=Ti4BPiEr1

The third term of Equation (9) can be transformed into:

(12) d4BTiTS4XSi=1000(XBi)z−(XBi)y010−(XBi)z0(XBi)x001(XBi)y−(XBi)x0dx2dy2dz2δx2δy2δz2

Assuming that the first 3×6 matrix in Equation (12) is $Q_{i}$ and the second 6×1 matrix is $\Delta X_{i}$, then Equation (12) can be transformed into:

(13) d4BTiTS4XSi=QiΔXi

In Equation (13), $\Delta X_{i}=M_{i}Er_{2}$, where $M_{i}$ represents a Jacobian matrix of 6×30 and $Er_{2}$ represents an error term of 30×1, and then Equation (13) can be transformed into:

(14) $$d_{4}^{B}T_{i}{}_{S}^{4}TX_{Si}=Q_{i}M_{i}Er_{2}$$

According to Equations (9), (11) and (14), we can get:

(15) $$X_{Bi}^{R}=X_{Bi}+{}_{4}^{B}T_{i}P_{i}Er_{1}+Q_{i}M_{i}Er_{2}$$

The matrix form of Equation (15) is:

(16) $$X_{Bi}^{R}-X_{Bi}=\left[\begin{array}{cc} {}_{4}^{B}T_{i}P_{i} & Q_{i}M_{i} \end{array}\right]\left[\begin{array}{c} Er_{1}\\ Er_{2} \end{array}\right]$$

Equation (16) is the detection error model of the robot vision system. The parameters to be calculated in the model are six differential translations/rotations $Er_{1}$ and 20 robotic MDH kinematic model parameter deviation $Er_{2}$.

Step 2. Solve the error compensation equations. Assuming $G_{i}=\left[\begin{array}{cc} {}_{4}^{B}T_{i}P_{i} & Q_{i}M_{i} \end{array}\right]$, $H_{i}=X_{Bi}^{R}-X_{Bi}$, and $\Delta E=\left[\begin{array}{c} Er_{1}\\ Er_{2} \end{array}\right]$, then when there are *m* centre positions, and each centre position is detected *n* times, we can get the equation:

(17) $$G=\left[\begin{array}{c} G_{11}\\ G_{12}\\ \cdots \\ \begin{array}{c} G_{1n}\\ \cdots \\ \begin{array}{c} G_{m1}\\ \cdots \\ G_{mn} \end{array} \end{array} \end{array}\right]=\left[\begin{array}{c} H_{11}\\ H_{12}\\ \cdots \\ \begin{array}{c} H_{1n}\\ \cdots \\ \begin{array}{c} H_{m1}\\ \cdots \\ H_{mn} \end{array} \end{array} \end{array}\right]\left[\begin{array}{c} Er_{1}\\ Er_{2} \end{array}\right]=H\Delta E$$

Solve Equation (17) using the SVD method:

(18) $$\Delta E=H^{+}G=VS^{+}U^{T}G$$

In Equation (18), $H^{+}$ is the generalised inverse matrix of matrix *H* after setting the error tolerance.

After obtaining the detection error compensation amount ΔE of the grinding robot vision system, we can calculate the compensated transformation matrix between the scanner frame and the robot base frame.

## 4. Data Processing of the Grinding Robot 3D Vision System

**Figure 2:**
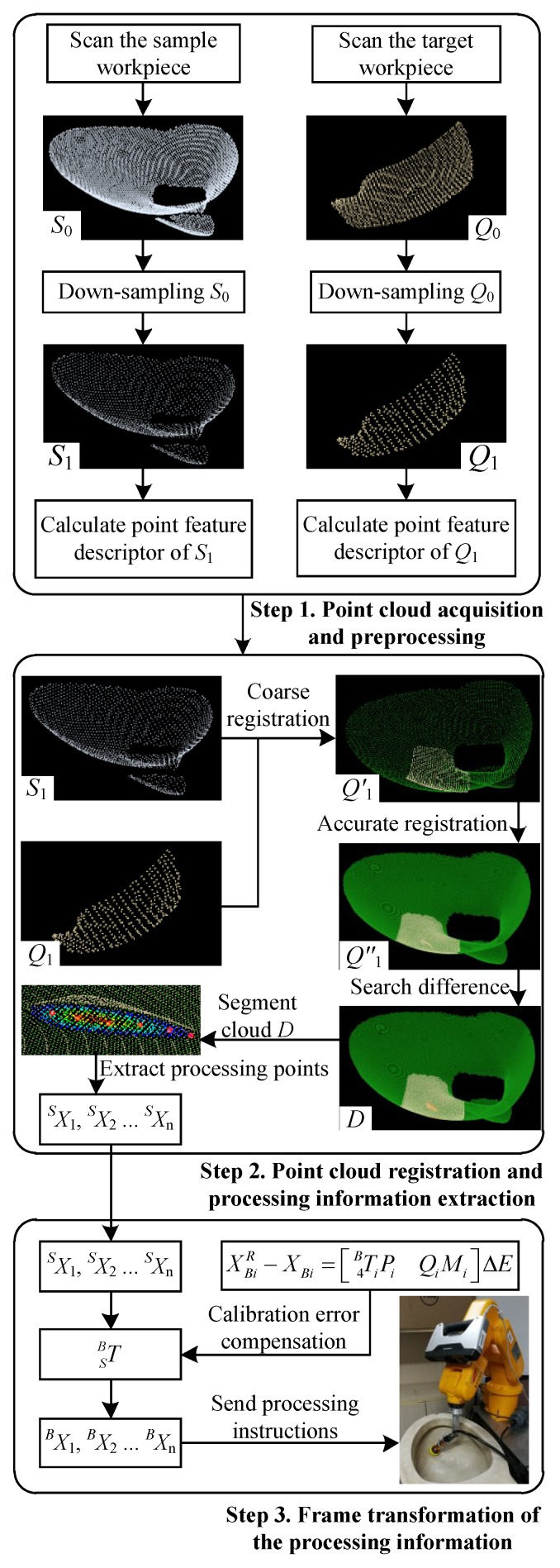
Procedure of data processing of the grinding robot 3D vision system.

Section 3 introduces the mathematical basis of the grinding robot 3D vision system. In this section, the data processing process for detecting the machining target using the 3D vision system is described, as shown in Figure 2.

It can be seen from Figure 2 that the detailed procedure of the data processing of the grinding robot 3D vision system can be described as follows:

Step 1. Point cloud acquisition and preprocessing

- Scan the sample workpiece (qualified workpiece) to get the sample point cloud $S_{0}$, and scan the target workpiece (workpiece to be processed) to get the test point cloud $Q_{0}$.
- Use the voxel grid filter to downsample the sample point cloud $S_{0}$ and the test point cloud $Q_{0}$ to get the point clouds $S_{1}$ and $Q_{1}$, respectively.
- Calculate the point feature descriptor of point clouds $S_{1}$ and $Q_{1}$ respectively, and obtain their respective FPFH.

Step 2. Point cloud registration and machining information extraction

- Use the SAC-IA to register the point clouds $S_{1}$ and $Q_{1}$ coarsely and obtain the paired point cloud pair $Q_{1}^{\prime }$.
- Use the ICP algorithm to accurately register the point cloud pair $Q_{1}^{\prime }$, resulting in the registered point cloud pair $Q_{1}^{\prime \prime }$.
- Use FLANN to search for difference point cloud *D*.
- Divide the difference point cloud *D*, then calculate the centroid of each grid, and search from the point cloud $Q_{1}$ for the points closest to these centroids as machining path points ${}^{S}X_{1}$, ${}^{S}X_{2}$ ⋯ ${}^{S}X_{n}$.

Step 3. Frame transformation of machining information

- Construct a detection error compensation model for the robot vision system, as shown in Equation (17).
- Obtain the calibration error ΔE of the grinding robot 3D vision system by solving Equation (18) with multiple points, and then calculate the transformation matrix ${}_{S}^{B}T$ between the compensated scanner frame and the robot base frame.
- The machining path points ${}^{S}X_{1}$, ${}^{S}X_{2}$ ⋯ ${}^{S}X_{n}$ obtained in Step 2 are converted into machining path points ${}^{B}X_{1}$, ${}^{B}X_{2}$ ⋯ ${}^{B}X_{n}$ described by the robot base frame, and then send the machining information commands to the grinding robot controller.

## 5. Experimental Results and Discussion

This section will build a grinding robot 3D vision system based on Section 2, Section 3 and Section 4 for related experiments. The experiments to be carried out in this section include: measurement accuracy experiment of the 3D vision system, calibration and calibration accuracy experiment of the grinding robot 3D vision system, machining target detection experiment using the grinding robot 3D vision system, and the effect of compound error of the grinding robot on detection accuracy of the 3D vision system.

### 5.1. Measurement Accuracy Experiment of the 3D Vision System

**Figure 3:**
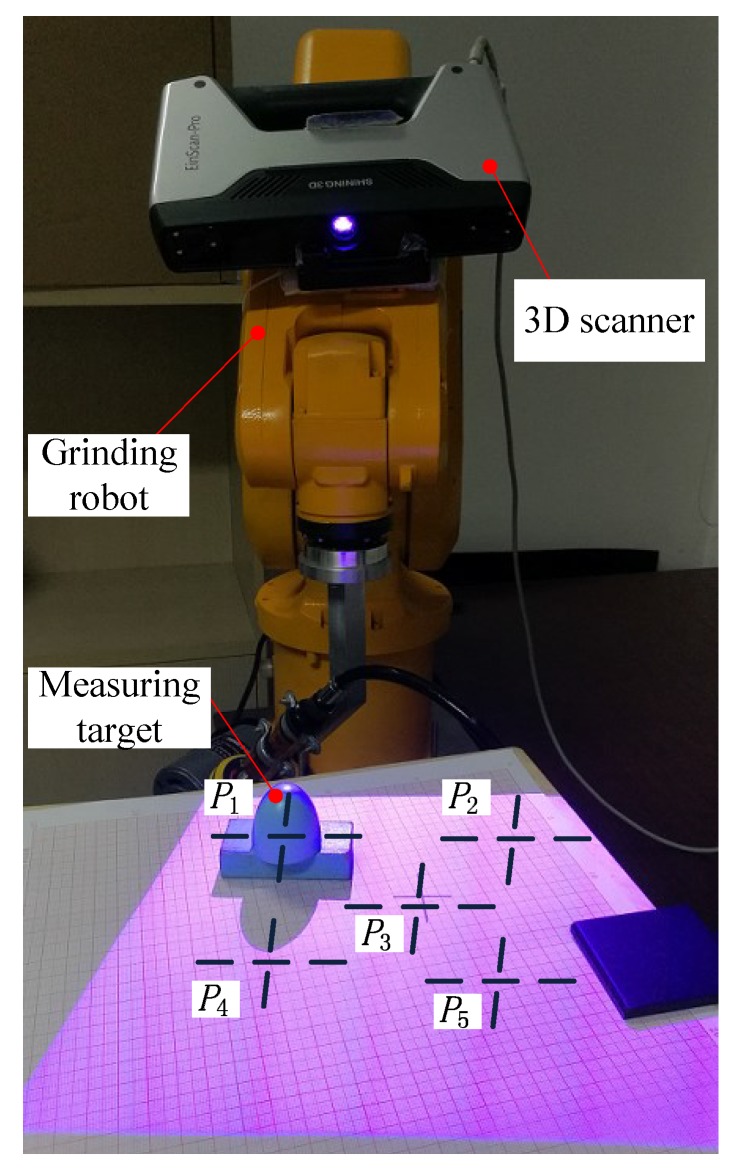
Experimental scenario for measuring the measurement accuracy of the 3D vision system.

The purpose of this experiment (Experiment 1) is to test the measurement accuracy of the 3D vision system proposed in this paper. The experimental scenario is shown in Figure 3. The grinding robot used here is manufactured by EFORT Intelligent Equipment Co., Ltd (Wuhu, China), the product model is ER3A-C60, the load is 3 kg, the working range is 1.256 m, and the repeat positioning accuracy is ±0.02 mm. The 3D scanner used here is manufactured by SHINING 3D TECH. (Hangzhou, China), the product model is EinScan-Pro, the fixed scanning accuracy is 0.05 mm, the measuring distance is 350 mm–450 mm, the nominal size of the 3D scanner’s field of view is 210 mm × 150 mm at the best measurement distance, and the scanner is fixed on the robot’s fourth joint and does not interfere with the regular rotation of all robot joints.

In the experiment, we placed a measuring target with a constant height (30.40 mm) at five different positions in the measurement space. The procedure of the experiment is as follows: Step 1. Adjust the orientation of the grinding robot so that the test bench is in the measuring space of the robot 3D vision system. Step 2. Keep the orientation of the grinding robot constant, and place the sample workpieces in five positions in the measurement space, as shown in Figure 3, $P_{1}$, $P_{2}$, $P_{3}$, $P_{4}$, and $P_{5}$. The height of the sample workpiece was measured using the 3D vision system proposed in this paper, and the experimental results are shown in Table 1.

After analysing the experimental results in Table 1, it can be found that the 3D vision system of the grinding robot has high measurement accuracy and measurement stability. When the theoretical measurement accuracy of the scanner in the free-scan mode is 0.05 mm, the absolute average error of the measurement result can reach 0.0277 mm. It is worth noting that the choice of the workpiece’s measuring distance is critical. In Figure 3, the positions of $P_{1}$, $P_{2}$, $P_{3}$, $P_{4}$, and $P_{5}$ are regularly placed, where $P_{3}$ coincides with the centre cursor of the vision sensor, and $P_{1}$, $P_{2}$, $P_{4}$, and $P_{5}$ are evenly distributed around it. Therefore, when using the 3D vision system, it is necessary to adjust the orientation of the robot based on the position of the workpiece to obtain higher measurement accuracy. The appropriate measurement distance can be found in the manual of the 3D scanner.

### 5.2. Calibration and Calibration Accuracy Experiment of the Grinding Robot 3D Vision System

**Figure 4:**
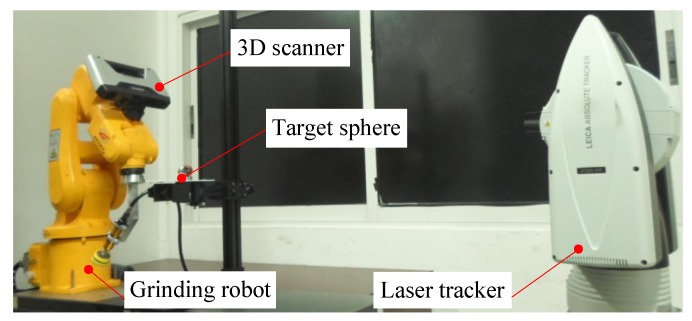
The calibration experiment platform for the vision measurement system of the grinding robot.

The calibration experiment platform for the vision measurement system of the grinding robot is shown in Figure 4, including a grinding robot, a 3D scanner, a target sphere, and a laser tracker. The grinding robot and the 3D scanner used here are the same as in Experiment 1. Moreover, the laser tracker used here is manufactured by Hexagon Manufacturing Intelligence, and the model number is Leica AT960-MR (Hexagon Manufacturing Intelligence, Unterentfelden, Switzerland). It is a laser measurement system with a powerful dynamic six-degrees-of-freedom (6DoF) function with a measurement accuracy of ± 15 µm + 6 µm/m when measured with a reflective ball, and optical centring accuracy is ±3 µm. The target sphere used here is a standard reflection sphere used with the Leica AT960-MR laser tracker.

The purpose of this experiment (Experiment 2) is to verify the coarse calibration method proposed in Section 3.7.

According to Equation (9), we can get the initial transformation matrix between the scanner’s frame and the robot’s fourth joint frame is:

$${}_{S}^{4}T_{initial}={}_{4}^{B}T^{-1}P_{B}P_{S}^{-1}=\left[\begin{array}{cccc} 0.0233 & -0.9996 & 0.0143 & 95.8105\\ 0.9918 & 0.0249 & 0.1257 & -96.9036\\ -0.1260 & 0.0113 & 0.9920 & -99.0726\\ 0 & 0 & 0 & 1.000 \end{array}\right]$$

We divide the measurement space into three layers evenly in the height direction, and then regularly choose three positions from each layer. When measuring the spherical coordinates of the target sphere placed at each position, the 3D vision system is performed from six different directions, thus obtaining 54 equations as shown in Equation (17). The detection error compensation amount can be obtained by solving the equations shown in Equation (18) by using the method of Equation (19). After accurate calibration, the coordinate transformation matrix of the scanner frame to the robot’s fourth joint frame is:

$${}_{S}^{4}T=\left[\begin{array}{cccc} 0.0259 & -0.9995 & 0.0158 & 97.1918\\ 0.9913 & 0.0277 & 0.1290 & -100.3302\\ -0.1294 & 0.0124 & 0.9915 & -97.8178\\ 0 & 0 & 0 & 1.0000 \end{array}\right]$$

The error compensation amount of the MDH parameter is shown in Table 2.

**Figure 5:**
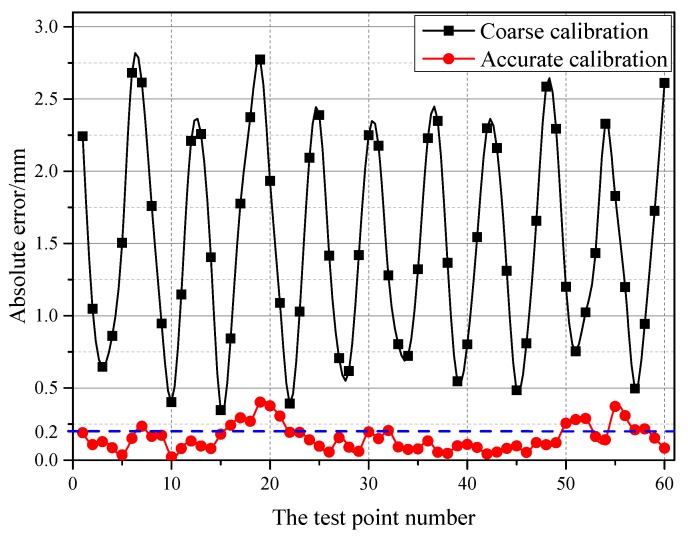
Comparison of the absolute error of the measurement results of the visual system after coarse and accurate calibration.

To verify the calibration accuracy of the grinding robot 3D vision system after accurate calibration, this experiment (Experiment 3) randomly selects ten target positions from the measurement space. Specifically, the ten positions in this experiment are distributed on the intermediate layer between any two layers in Experiment 2 because the theoretical measurement error is the largest here. The grinding robot 3D vision system measures the spatial position of the target sphere from six different directions. The statistical results of the Euclidean distance between the actual coordinates of the centre of the target sphere and the measured coordinates obtained by the 3D vision system are shown in Figure 5.

It can be seen from Figure 5 that the accuracy of the vision system has been dramatically improved after accurate calibration. Analyzing the above data, we can get the following conclusions: (1) The maximum deviation after accurate calibration is 0.402 mm, the average absolute error is 0.154 mm, and the standard deviation is 0.091. (2) The average absolute error of the Euclidean distance between the actual coordinate of the measurement space and the measured coordinate is mostly within 0.2 mm, and the maximum error is mostly within 0.3 mm. This detection error can meet the general accuracy requirements of the grinding operation.

### 5.3. Machining Target Detection Experiment Using the Grinding Robot 3D Vision System

**Figure 6:**
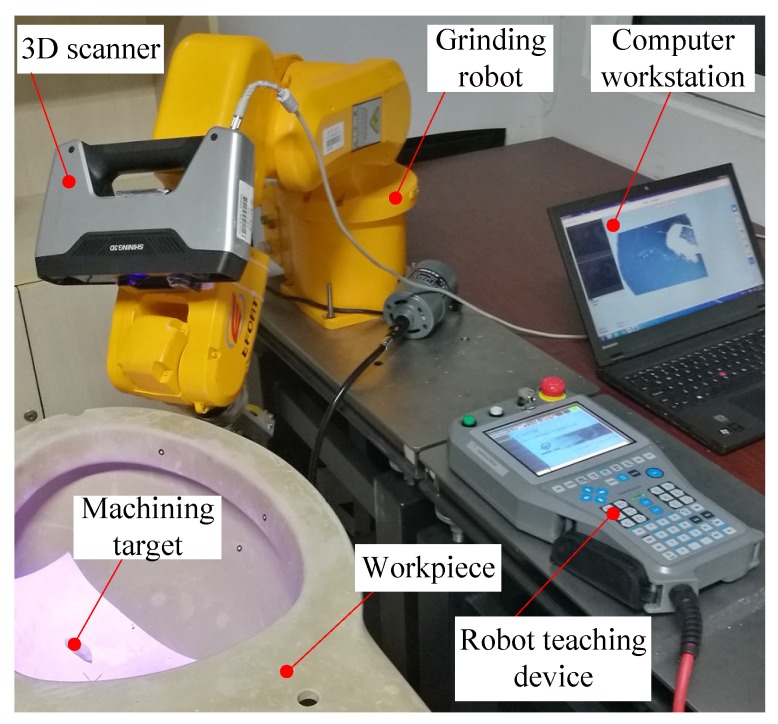
Experimental scenario for detecting the machining target using the 3D vision system.

**Figure 7:**
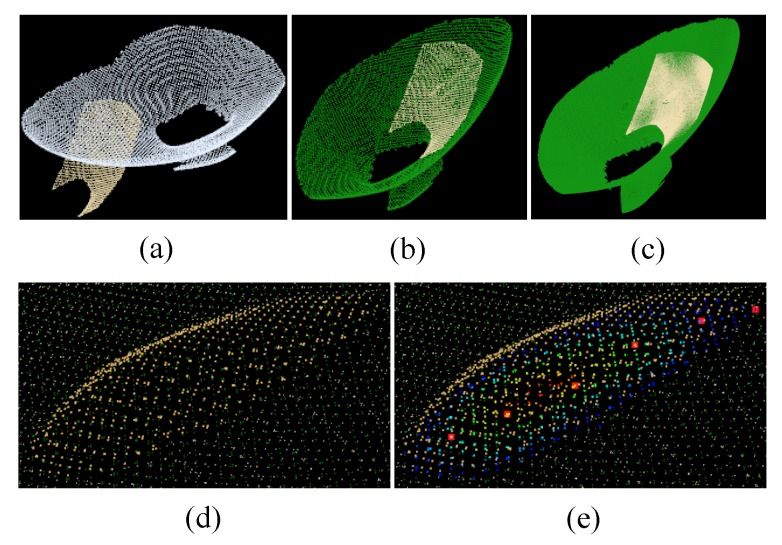
Point cloud and machining path point information in the detection process of the machining target. (**a**) Unregistered point cloud pair; (**b**) Coarse registration; (**c**) Accurate registration; (**d**) Difference point cloud; (**e**) Machining path points.

The purpose of this experiment (Experiment 4) is to test the effectiveness of the grinding robot 3D vision system proposed in this paper for the detection of the machining target. The experimental scenario is shown in Figure 6. The grinding robot and the 3D scanner used here are the same as in Experiment 1. According to the data processing procedure of the grinding robot 3D vision system shown in Figure 2, machining path points of the machining target can be obtained, and the point cloud and machining path point information of the intermediate process is shown in Figure 7. The detected machining path points described using the scanner frame are shown in Table 3, and the machining path points described using the robot base frame after the frame transformation are shown in Table 4.

Table 4 shows the machining information output from the 3D vision inspection system of the grinding robot. The machining path points in Table 3 and Table 4 are the machining information described here. The final output machining information is the coordinate and normal vector of the machining path points in the robot base frame. The grinding robot carries the grinding tool through these path points to complete the grinding task of the machining target. It is worth noting that the orientation of the grinding tool in the grinding process is opposite to the direction of the normal vector at each machining path point. Besides, the machining target information output in this experiment contains six machining path points, which is caused by the parameter setting of the segmentation difference point cloud in the data processing process of the 3D vision system. The more grids after the difference point cloud are divided, the more machining path points will be obtained. Of course, the setting of the grid size is not only related to the processing accuracy of the workpiece but also related to the processing technology.

### 5.4. The Effect of the Compound Error of the Grinding Robot on the Detection Accuracy of the 3D Vision System

**Figure 8:**
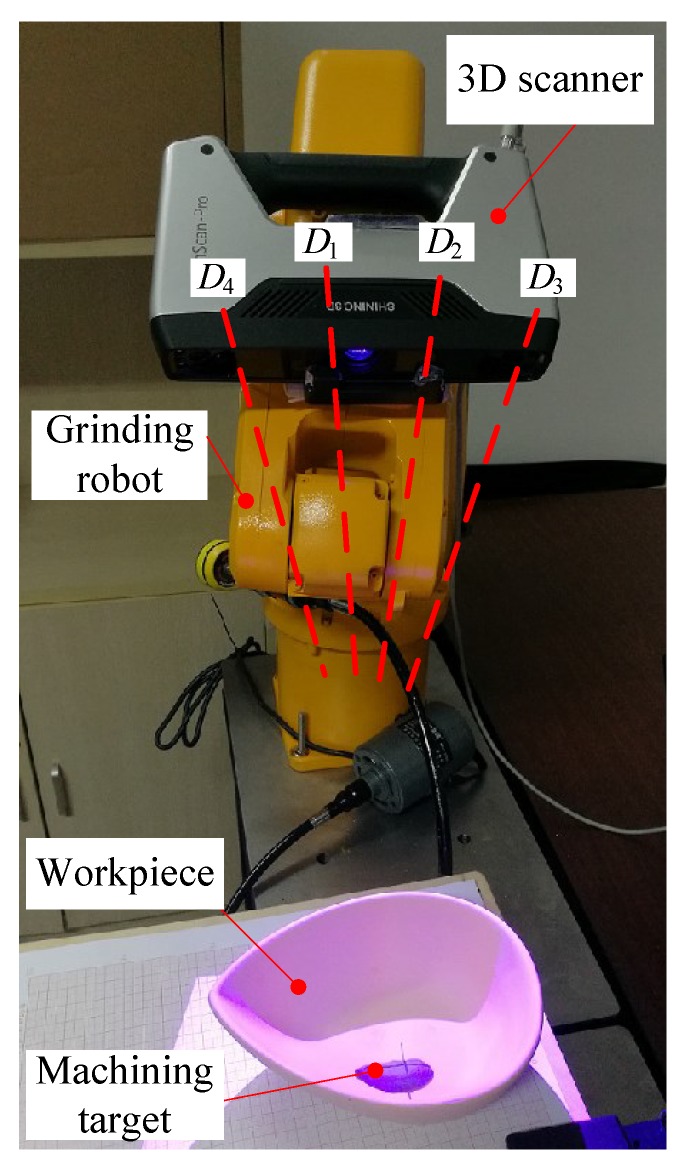
Experimental scenario of the effect of the compound error on 3D vision system detection accuracy.

**Figure 9:**
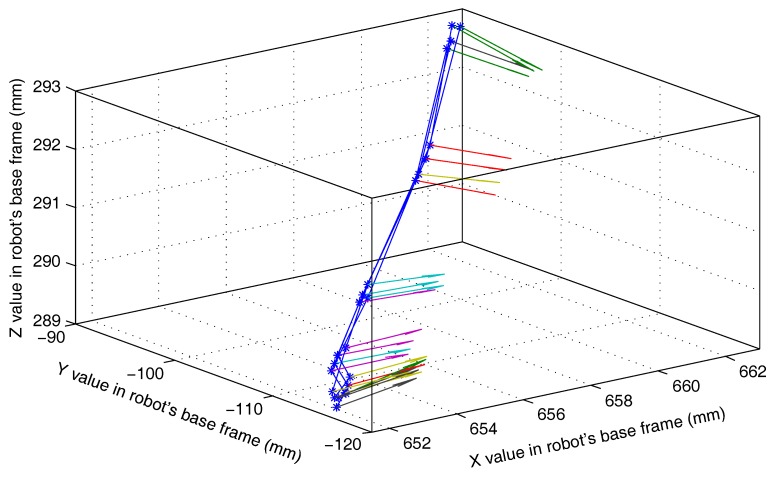
Machining paths obtained by the grinding robot 3D vision system with different orientations.

**Figure 10:**
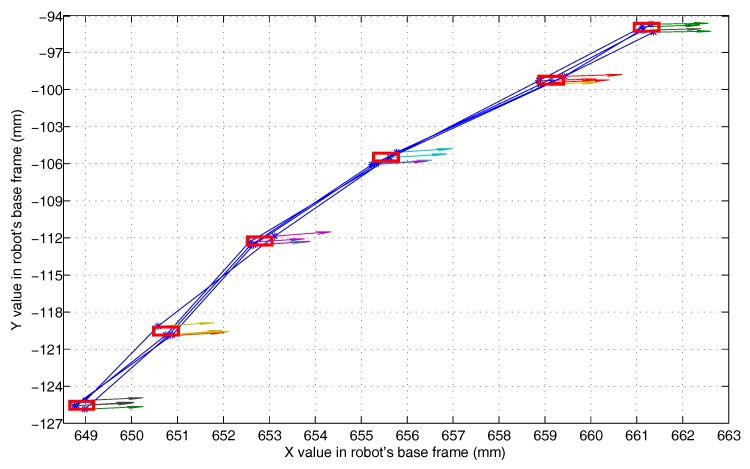
The projection of the paths shown in Figure 9 on the XY plane.

**Figure 11:**
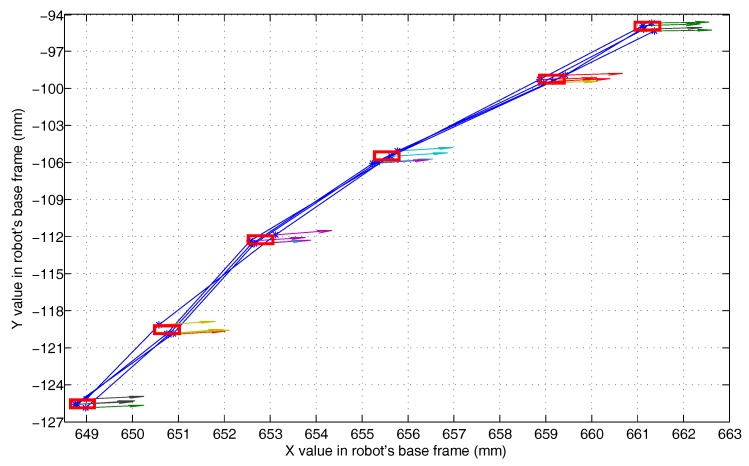
The projection of the paths shown in Figure 9 on the XZ plane.

**Figure 12:**
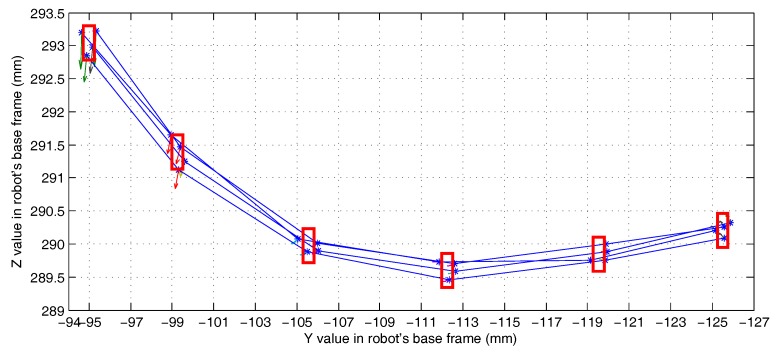
The projection of the paths shown in Figure 9 on the YZ plane.

This experiment (Experiment 5) is used to test the effect of the compound error caused by the scanner measurement error, the point cloud registration error, the robot motion error, and the visual system calibration error on the detection accuracy of the 3D vision system. The experimental scenario is shown in Figure 8. The grinding robot and the 3D scanner used here are the same as in Experiment 1. In the experiment, we fix the workpiece in the same position in the measurement space. The grinding robot 3D vision system detects the machining target on the workpiece from four different orientations $D_{1}$, $D_{2}$, $D_{3}$, and $D_{4}$, respectively, and extracts the corresponding machining path points as machining information. Figure 9 is the curves (machining paths) obtained by connecting machining path points in the robot base frame. Figure 10, Figure 11 and Figure 12 are the projections of the curves shown in Figure 9 on the XY, XZ, and YZ plane, respectively.

It can be seen from Figure 9 that the four curves (machining paths) are almost coincident. To study the discreteness of the machining path points on the curve, we project the four curves of Figure 9 on the XY, XZ, and YZ plane respectively to obtain Figure 10, Figure 11 and Figure 12. In Figure 10, Figure 11 and Figure 12 we can see that there are six machining path points on each curve. To see the discreteness of the corresponding machining path points in the three directions X, Y, and Z, we use the squares with a side length of 0.5 mm to surround the machining path point in Figure 10, Figure 11 and Figure 12. From Figure 10, Figure 11 and Figure 12, it can be found that the corresponding machining path points can be surrounded by the square with a side length of 0.5 mm. This proves that the 3D vision system proposed in this paper has high stability of repeated detection, and the absolute measurement accuracy of the 3D vision system caused by the compound error is about 0.25 mm. Such detection accuracy can satisfy the grinding of the workpiece with general accuracy requirements because the radius of a grinding tool is usually much larger than the detection error. It is worth noting that: (1) Figure 10, Figure 11 and Figure 12 show that squares with a side length of 0.5 mm are rectangular because the scale of the axes is different. (2) The arrows with different colours shown in Figure 9, Figure 10, Figure 11 and Figure 12 indicate the normal direction of the machining path points detected by the 3D vision system, but this paper does not accurately measure the error in the normal direction because on the one hand, the normal direction of the machining path point is difficult to measure, and on the other hand, the normal measurement error is usually positively correlated with the measurement error of the corresponding point position.

## 6. Conclusions

This paper proposes a 3D vision system for grinding robots, which is used to automatically detect the machining target on the workpiece and convert the machining information to the base frame of the grinding robot. Its key point is to obtain machining information by comparing and analysing the difference between the test point cloud and the sample point cloud. Also, the frame transformation matrix used to transform the machining information described in the scanner frame into the robot base frame is compensated.

The experimental results show that the proposed 3D vision system can not only effectively extract the machining path points on the machining target but also to transform it into the robot base frame. Besides, the average absolute error of the height measurement of the same workpiece at different positions is 0.0277 mm. The absolute average error of repeated measurements of the target sphere’s centre position at different locations using the 3D vision system is 0.154 mm with a standard deviation of 0.091. The absolute measurement error of the 3D vision system caused by the compound error (including scanner measurement error, point cloud registration error, robot motion error, and vision system calibration error) is usually less than 0.25 mm. Such detection accuracy can satisfy the grinding of the workpiece with general accuracy requirement. Besides, the proposed 3D vision system is simple in structure and can be easily integrated into an intelligent grinding system, so it is suitable for industrial sites.

The grinding robot 3D vision system presented in this paper is suitable for the automatic detection of the machining target of the workpiece with general accuracy requirements. However, the registration accuracy of the point cloud pair will affect the detection accuracy. Besides, the extraction of the machining path point is related to the processing technology. Therefore, in the future, the effect of the point cloud pair’s registration accuracy and processing technology on the 3D vision system measurement accuracy may be studied.

References

## Figures and Tables

**Table 1:** Experimental results of measuring the measurement accuracy of the 3D vision system.

Position Number	$P_{1}$	$P_{2}$	$P_{3}$	$P_{4}$	$P_{5}$
Measured height (mm)	30.3999	30.4026	30.4522	30.4175	30.4662
Absolute error (mm)	0.0001	0.0026	0.0522	0.0175	0.0662

**Table 2:** The error compensation amount of the MDH parameter.

*i*	$\Delta \alpha_{i-1}$ (°)	$\Delta a_{i-1}\left(\mathrm{mm}\right)$	$\Delta d_{i}\left(\mathrm{mm}\right)$	$\Delta \theta_{i}$ (°)	$\Delta \beta_{i}$ (°)
1	−0.0088	−1.0167	−3.1596	0.0052	0
2	−0.0001	0.0001	−1.0152	−0.0007	0
3	0.0022	−0.0007	0.3185	0.0074	−0.0018
4	−0.0027	1.0445	0.0834	0.0026	0

**Table 3:** Machining path points in the scanner frame detected by the 3D vision system.

Machining Path Point Number	PS(x/y/z) (mm)	NS(x/y/z) (rad)
1	2.9196/−16.9633/439.4346	0.6721/−0.2216/−0.7066
2	5.8379/−14.1931/437.0458	0.6734/−0.2014/−0.7113
3	8.5740/−8.8756/432.9619	0.6762/−0.1792/−0.7146
4	12.8166/−4.4634/428.9439	0.6869/−0.1583/−0.7093
5	16.8304/−0.7351/425.3878	0.6862/−0.1520/−0.7114
6	22.3266/2.7024/421.6943	0.6850/−0.1405/−0.7149

**Table 4:** Machining path points in the robot base frame after frame transformation.

Machining Path Point Number	PB(x/y/z) (mm)	NB(x/y/z) (rad)
1	552.5296/−596.9379/−46.6984	0.8525/−0.1176/1.7602
2	548.0370/−596.0266/−45.7583	0.8463/−0.0979/1.7351
3	541.6790/−592.6831/−44.8424	0.8415/−0.0759/1.7068
4	534.7713/−590.8615/−43.2371	0.8451/−0.0547/1.6779
5	528.5730/−589.5172/−41.6772	0.8426/−0.0486/1.6705
6	521.4130/−589.2253/−39.5995	0.8383/−0.0373/1.6571
